# The cannabinoid ligand LH-21 reduces anxiety and improves glucose handling in diet-induced obese pre-diabetic mice

**DOI:** 10.1038/s41598-017-03292-w

**Published:** 2017-06-21

**Authors:** Silvana Y. Romero-Zerbo, Inmaculada Ruz-Maldonado, Vanesa Espinosa-Jiménez, Alex Rafacho, Ana I. Gómez-Conde, Lourdes Sánchez-Salido, Nadia Cobo-Vuilleumier, Benoit R. Gauthier, Francisco J. Tinahones, Shanta J. Persaud, Francisco J. Bermúdez-Silva

**Affiliations:** 1 Unidad de Gestión Clínica Intercentros de Endocrinología y Nutrición, Instituto de Investigación Biomédica de Málaga (IBIMA), Hospital Regional Universitario de Málaga, Universidad de Málaga, Málaga, Spain; 2 Centro de Investigación Biomédica en Red de Diabetes y Enfermedades Metabólicas Asociadas (CIBERDEM), Málaga, Spain; 3 Diabetes Research Group, Division of Diabetes & Nutritional Sciences, Faculty of Life Sciences & Medicine, King’s College London, Guy’s Campus, London, SE1 1UL UK; 4 Department of Physiological Sciences, Center of Biological Sciences, Federal University of Santa Catarina (UFSC), 88040-900 Florianópolis, SC Brazil; 5 Bioimaging facility, Instituto de Investigación Biomédica de Málaga (IBIMA), Hospital Regional Universitario de Málaga, Universidad de Málaga, Málaga, Spain; 6 Stem Cells Department, Andalusian Center for Molecular Biology and Regenerative Medicine (CABIMER), Seville, Spain; 7 Centro de Investigación Biomédica en Red de Obesidad y Nutrición (CIBEROBN), Málaga, Spain

## Abstract

LH-21 is a triazol derivative that has been described as a low-permeant neutral CB1 antagonist, though its pharmacology is still unclear. It has been associated with anti-obesity actions in obese rats. However, its role in preventing type 2 diabetes (T2D) onset have not been studied yet. Given CB1 receptors remain as potential pharmacological targets to fight against obesity and T2D, we wanted to explore the metabolic impact of this compound in an animal model of obesity and pre-diabetes as well as the lack of relevant actions in related central processes such as anxiety. C57BL/6J mice were rendered obese and pre-diabetic by feeding a high-fat diet for 15 weeks and then treated with LH-21 or vehicle for two weeks. Food intake, body weight and glucose handling were assessed, together with other relevant parameters. Behavioural performance was evaluated by the open field test and the elevated plus maze. LH-21 did not affect food intake nor body weight but it improved glucose handling, displaying tissue-specific beneficial actions. Unexpectedly, LH-21 induced anxiolysis and reverted obesity-induced anxiety, apparently through GPR55 receptor. These results suggest that LH-21 can be a new candidate to fight against diabetes onset. Indeed, this compound shows potential in counteracting obesity-related anxiety.

## Introduction

Obesity and related co-morbidities are reaching pandemic proportions worldwide. In 2014, 11% of men and 15% of women age 18 and older were obese, while more than 42 million children under the age of five years were overweight in 2013^1^. The leading risk factors for type 2 diabetes (T2D) are excess body weight and physical inactivity, and T2D is highly correlated with the global prevalence of obesity, which has nearly doubled since 1980^1^. Currently obesity-related diseases constitute a heavy burden for health systems, so it is not surprising there is an increasing demand for innovative therapeutic intervention to alleviate these disabling conditions.

Since the discovery of the anorexigenic actions of cannabinoid CB1 receptor blockade and the beneficial impact it has on body weight homeostasis and metabolic control, there has been an intense basic and clinical research effort aiming at developing and marketing new anti-obesity agents based on this receptor^2^.

The prototypical molecule of this type of compound is rimonabant, which was in fact marketed in Europe and many other countries in 2006 as an anti-obesity treatment (Acomplia®), as an adjunct to diet and exercise for the treatment of obese patients or overweight patients with associated risk factors. However, due to psychiatric side effects, rimonabant was withdrawn from the market in 2009^2, 3^. Interestingly, soon afterwards it became evident that blockade of central CB1 receptors was not required to achieve metabolic benefits^4, 5^, and the development of a second generation of CB1-based drugs focused on peripheral antagonism was intensified^6^. Over the last few years several peripheral-acting CB1 antagonists as well as other cannabinoid-based agents have been described. Examples are the compounds AM6545^7^, JD5037^8^, TM38837^9^ and LH-21^10^.

LH-21, 5-(4-chlorophenyl)-1-(2,4-dichlorophenyl)-3-hexyl-1H-1,2,4-triazole, was initially described as an *in vivo* CB1 antagonist with a paradoxic low affinity *in vitro* for CB1 receptors, low penetration into the brain and being devoid of inverse agonist properties^10^. However, its pharmacology isn’t clear and other authors have reported extra-CB1 effects of LH-21 and its ability in crossing the blood-brain-barrier (BBB)^11^. This compound has been found to decrease feeding behaviour in food-deprived rats and to reduce food intake and body weight gain in a genetic model of obesity, the obese Zucker rat^12^. Furthermore, it reduced feeding and body weight gain in Wistar rats fed a high-fat diet^13^. However, the beneficial actions of this compound in diabetes onset as well as the putative mechanisms involved have yet to be clarified. Moreover, despite this compound being able to cross the BBB to some extent, its putative modulatory actions of obesity-related anxiety are unknown.

To address these unexplored areas we have treated a mouse model of diet-induced obesity and pre-diabetes for two weeks with LH-21 (3 mg/Kg). Body weight gain, food intake, glucose homeostasis, systemic inflammatory markers as well as pancreas and liver histopathology were studied. In addition, a behavioural study of the effects of LH-21 on obesity-related anxiety was performed.

## Results

### LH-21 improves glucose handling in obese pre-diabetic mice

**Figure 1:**
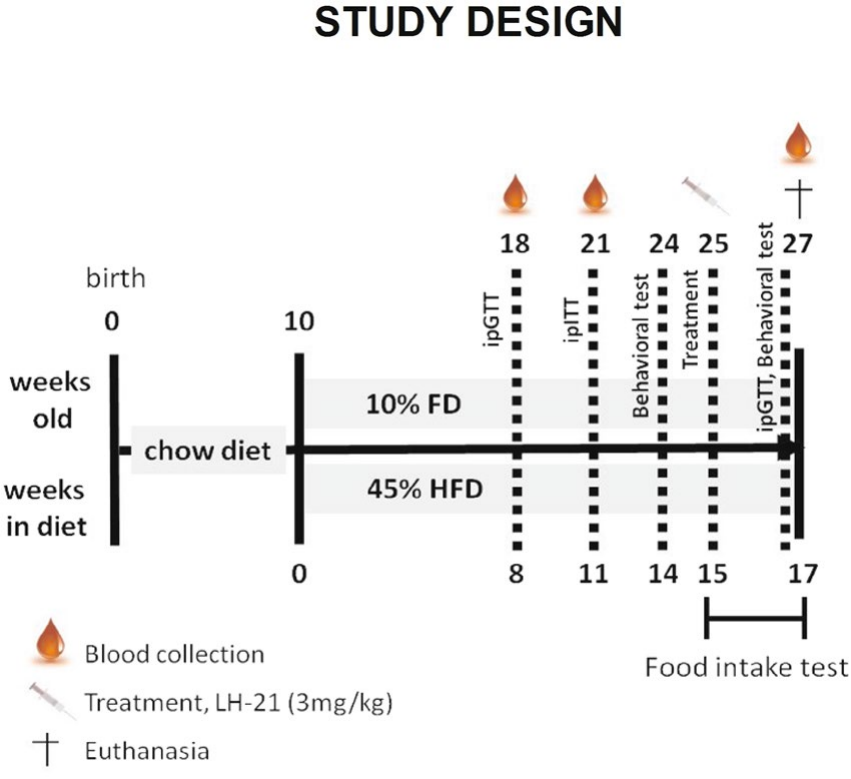
Study design. Outline representing the design of the present study. Drawings are under Creative Commons license CC0 from Pixabay.com.

**Figure 2:**
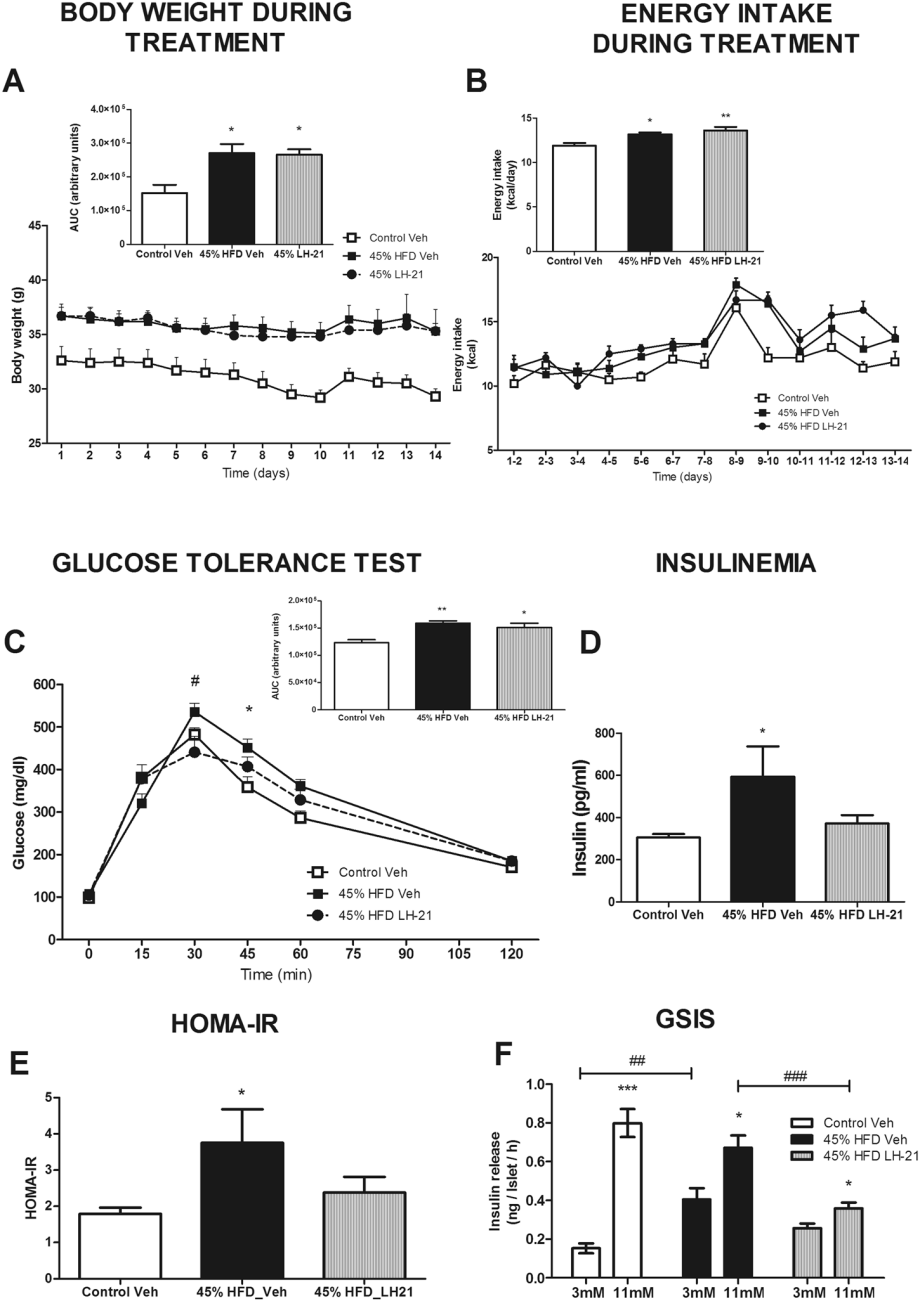
Metabolic characterization of obese pre-diabetic LH-21-treated mice. (**A**) Body weight changes during the two weeks of treatment with 3 mg/Kg LH-21. Body weight was not affected by the treatment and both vehicle- and LH-21-injected mice remained obese when compared to the control group. (**B**) Food intake was monitored daily, the line and the bar graphs represent the mean daily intake and the mean total intake of energy (Kcal), respectively, during the two week period. No changes in caloric intake was detected between vehicle- and LH-21-injected mice, both groups displaying a higher caloric intake than the control group. (**C**) Glucose tolerance was assessed by an i.p. GTT in fasted mice injected with 2 g/Kg glucose. AUC analysis for the entire time frame revealed glucose intolerance in both vehicle- and LH-21- injected mice, though lower plasma glucose levels were found in LH-21-injected mice at time point 30 minutes after glucose challenge (Student’s t test). (**D**) Insulin plasma levels in fasted mice as assessed by ELISA. 45% HFD-vehicle mice had higher insulinemia but not those treated with LH-21. (**E**) HOMA-IR was used as a subrrogate measure of insulin resistance (HOMA-IR = insulin (mU/l) × glucose (mmol/l)/22.5). 45% HFD-vehicle mice had higher HOMA-IR but not those treated with LH-21. (**F**) Glucose-stimulated insulin secretion from islets isolated from obese pre-diabetic mice injected with vehicle or LH-21. Islets isolated from LH-21-treated mice secreted less insulin than those injected with vehicle with this difference being particularly marked in the high glucose condition. (**A**–**E**) n = 8–10 mice each group. One-way ANOVA and Bonferroni’s post-test, *p < 0.05, **p < 0.01 *versus* control; ^#^p < 0.05 *versus* 45% HFD-Vehicle. (**F**) Islets from two different mice from each group, two independent experiments, n = 6 experimental wells for each condition, two-way ANOVA with Bonferroni’s post-test; *p < 0.05 *versus* 3 mM Glc, ^##^p < 0.01 and ^###^p < 0.001.

The present work was designed to evaluate the actions of the cannabinoid ligand LH-21 in diabetes prevention, with a focus on its effects on the pancreas and liver, and determine its effects on obesity-related anxiety. A general overview of the study design is depicted in Fig. 1. In brief, according the literature a mouse model of diet-induced obesity^14^ was used to assess the effects of subchronic treatment with LH-21 (3 mg/Kg/day for two weeks) on body weight, food intake, glucose homeostasis and inflammatory status, and a behavioural study was also performed on these animals. LH-21 dosage was selected according to previous works using this compound^12, 13, 15^. As expected, mice fed a high-fat-diet (HFD; 45% energy intake from saturated fat) became obese when compared to those fed a standard-fat diet (10% energy intake from fat), with differences in body weight clearly evident at week 11 of the dietary intervention (Supplementary Figure 1A), being close to 20% increase over control at week 15, as expected^16^. Moreover, impaired glucose tolerance and reduction in insulin sensitivity were detected after 8 and 11 weeks of HFD, respectively, with no changes in fasting glucose (Supplementary Figure 1B,C), suggesting alterations of glucose homeostasis compatible with a pre-diabetic stage. Obese pre-diabetic mice treated daily for up to 2-weeks with LH-21 (3 mg/Kg *b*.*w*.) did not display weight loss nor decreased food intake as compared to vehicle treated mice (Fig. 2A,B). They remained glucose intolerant when compared to vehicle-injected mice as assessed by glucose area-under-the-curve (AUC) calculation (ANOVA analysis for the entire time frame, Fig. 2C insert), though decreased glucose values were found at time point 30 min after glucose overload (Student’s t test), i.e. there was a lower plateau in glucose excursion (Fig. 2C). Furthermore, a tendency for LH-21 to decrease fasting glucose was found (Vehicle: 108 ± 4 mg/dL versus LH-21: 98 ± 7 mg/dL, p = 0.193 Student’s t test) and increased insulinemia and HOMA-IR (homeostatic model assessment -insulin resistance) were not present in LH-21-injected mice (Fig. 2D,E). Regarding endocrine pancreas function, islets isolated from HFD mice that had been treated for two weeks with LH-21 were submitted to glucose-stimulated insulin secretion (GSIS) experiments. Two-way ANOVA analysis revealed that islets from LH-21-treated mice showed a reduction in insulin release when compared to islets from vehicle-injected mice (Fig. 2F). Indeed, decreased secretion was found in response to 11 mM glucose in islets from mice treated with LH-21 (Fig. 2F, Bonferroni’s post-test). These findings, together with decreased insulinemia, decreased insulin resistance and lower glucose levels at specific time points during the glucose tolerance test (GTT) (Fig. 2C–E) suggest LH-21 improves glucose handling by favouring peripheral glucose uptake.

### LH-21 displays several anti-inflammatory and cytoprotective actions

**Figure 3:**
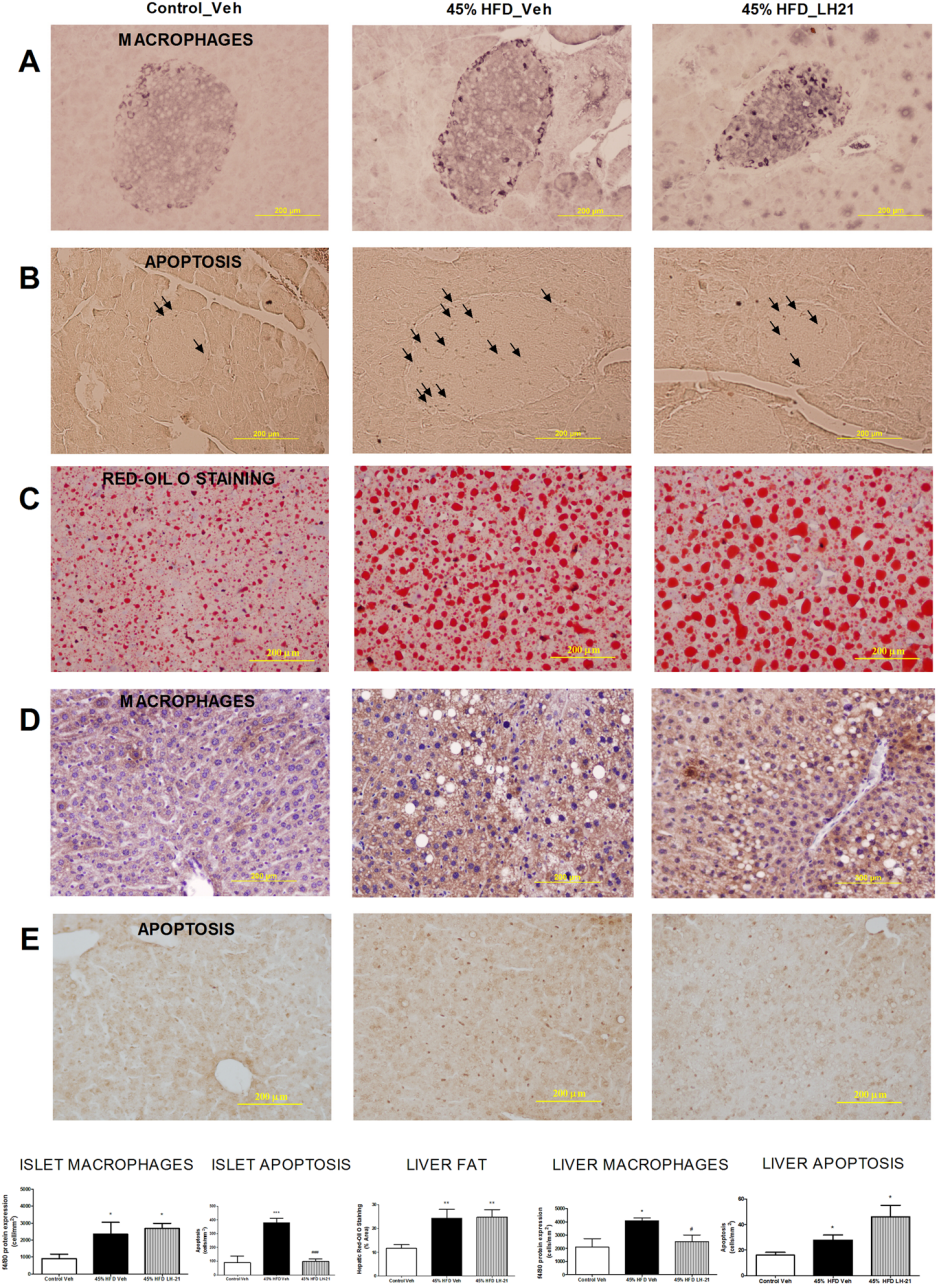
Effects of LH-21 in the islets of Langerhans and the liver. (**A**) Macrophage infiltration in the islets was assessed by immunohistochemistry with the specific marker F4/80. Quantification of immunostaining within islets revealed increased macrophage infiltration in 45% HFD mice, with no differences between vehicle- and LH-21-injected mice. The images are representative of islets from four mice each group and four different sections from each pancreas; one-way ANOVA and Bonferroni’s post-test, *p < 0.05 *versus* control. (**B**) Apoptosis in islet cells was assessed by a specific apoptosis kit. The number of apoptotic cells was increased in islets from 45% HFD-vehicle mice, while there was no increased apoptosis in islets from 45% HFD-LH-21 mice when compared to control mice. (**C**) Fat accumulation in the liver was studied by red-oil O staining of hepatic sections. Quantification of this specific marker revealed increased fatty content in the liver of 45% HFD mice, both vehicle- and LH-21-injected mice. (**D**) Macrophage infiltration in the liver was assessed by immunohistochemistry with the specific marker F4/80. These sections were counterstained with haematoxylin. Quantification of immunostaining in the liver revealed increased macrophage infiltration in 45% HFD-vehicle when compared to control-vehicle mice, but no increase was detected in 45% HFD-LH-21 mice. (**E**) Apoptosis in the liver was assessed by a specific apoptosis kit. The number of apoptotic cells was increased in the livers of both 45% HFD-vehicle and 45% HFD-LH-21 mice when compared to control-vehicle mice. (**A**,**B**) The images are representative of islets from four mice in each group and four different sections from each pancreas; one-way ANOVA and Bonferroni’s post-test, ***p < 0.001 *versus* control, ^###^p < 0.001 *versus* 45% HFD-vehicle. (C-E) The images are representative of livers from four mice in each group and four different sections from each liver; one-way ANOVA and Bonferroni’s post-test, *p < 0.05, **p < 0.01 *versus* control-vehicle, ^#^p < 0.05 *versus* 45% HFD-vehicle.

A panel of inflammatory cytokines was assayed on plasma samples from these mice together with ELISA measurement of the levels of the adipokines leptin and adiponectin (Table 1). Plasma leptin, high-molecular-weight (HMW) adiponectin, IL-6 and the chemokine (C-X-C motif) ligand 1 (CXCL1) levels were found to be negatively impacted in obese pre-diabetic mice, suggesting a systemic pro-inflammatory state (Table 1). Both leptin and CXCL1 levels were slightly decreased after LH-21 treatment, the latter not being statistically different when compared to the control group while no effect on adiponectin and IL-6 were detected. Moreover, no changes among groups were detected for IL-5, IL-2, IL-12p70, IL-10 and IFN-γ (Table 1). LH-21 treatment was not associated with a decrease in obesity-induced macrophage infiltration of the islets (Fig. 3A) and did not revert the obesity-induced decrease in M2 macrophages, as assessed by Mrc-1 and CD163 immunostaining (Supplementary Figure 3A,B). However, obesity-induced apoptosis in islets was reverted by LH-21 (Fig. 3B). In the liver, increased red-oil staining was observed in HFD-mice when compared to control mice, suggesting ectopic accumulation of fat (Fig. 3C). Indeed, both macrophage infiltration and apoptosis were increased in the livers of obese pre-diabetic mice (Fig. 3D,E). Macrophage infiltration was decreased in the liver by LH-21 (Fig. 3D) without modifying the levels of the M2 subtype (Supplementary Figure 3C,D), but neither liver steatosis nor liver apoptosis were ameliorated by LH-21 (Fig. 3C and E).

**Table 1:** Systemic inflammatory markers in obese pre-diabetic LH-21-treated mice.

Systemic Inflammatory Markers	CONTROL		45% HFD VEH		45% HFD LH-21	
**Leptin** (pg/ml)	4121 ± 749		19550 ± 8501**		13720 ± 7917*	
**HMWAdiponectin** (ng/ml)	5397 ± 2679		2864 ± 746*		2942 ± 1100*	
**IL-6** (pg/ml)	51,7 ± 13,0		211,4 ± 107,6*		183,4 ± 165,9*	
**CXCL1** (pg/ml)	137,8 ± 29,9		383,9 ± 209,8*		232,4 ± 169,5	
**IL-5** (pg/ml)	5,46 ± 1,78		8,99 ± 4,26		12,95 ± 11,67	
**IL-2** (pg/ml)	5,31 ± 1,59		5,20 ± 1,03		5,56 ± 1,46	
**IL-12 p70** (pg/ml)	61,41 ± 10,53		78,09 ± 19,51		95,73 ± 31,61	
**IL-10** (pg/ml)	23,23 ± 16,48		22,45 ± 11,86		26,11 ± 11,12	
**IFN-γ** (pg/ml)	1,31 ± 0,71		1,45 ± 0,44		1,77 ± 0,68	

The levels of leptin and HMW adiponectin in plasma were measured with a commercial ELISA. A mouse pro-inflammatory panel kit was used for the quantitative determination of IFN-γ, IL-2, IL-5, IL-6, CXCL1, IL-10 and IL-12p70 by multi-array electrochemiluminescence detection technology. n = 8–10 mice each group. One-way ANOVA and Bonferroni’s post-test, *p < 0.05, **p < 0.01 *versus* control.

### LH-21 promotes anxiolysis in obese pre-diabetic mice and reverts obesity-induced anxiety

**Figure 4:**
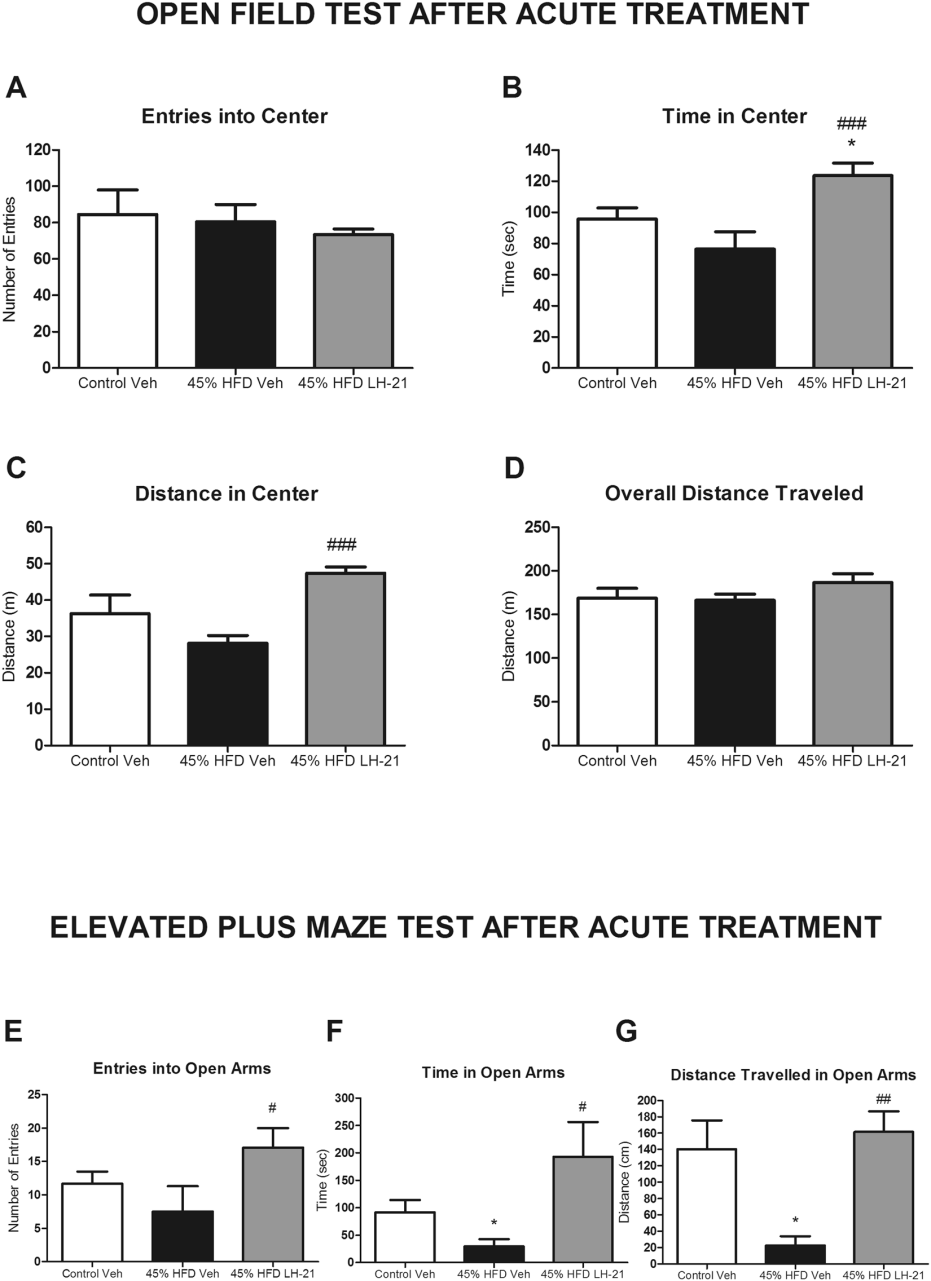
Behavioural study in obese pre-diabetic mice acutely treated with LH-21. (**A**–**D**) Exploratory activity was recorded for ten minutes in the open field maze in control, 45% HFD-vehicle and 45% HFD-LH-21 mice. Entries into center (**A**), time in center (**B**), distance in center (**C**) and overall distance travelled (**D**) were monitored. No changes were detected between control and 45% HFD-vehicle mice. However, acute LH-21 injections increased exploratory activity in the center in 45% HFD mice, as assessed by increased time and distance travelled in center; (**E**–**G**) Anxiety was analysed by the elevated plus maze test in these mice. Entries into open arm (**E**), time in open arms (**F**) and distance travelled in open arms (**G**) were recorded for five minutes. 45% HFD-vehicle mice showed an anxiety-like behaviour with decreased time in open arms and distance travelled in open arms when compared to control mice. Acute injections of LH-21 in 45% HFD mice increased the number of entries into open arms and reverted the HFD-induced decrease in time in open arms and distance travelled in open arms. n = 8–10 mice each group. One-way ANOVA and Bonferroni’s post-test, *p < 0.05 *versus* control; ^#^p < 0.05, ^##^p < 0.01 *versus* 45% HFD-Vehicle.

**Figure 5:**
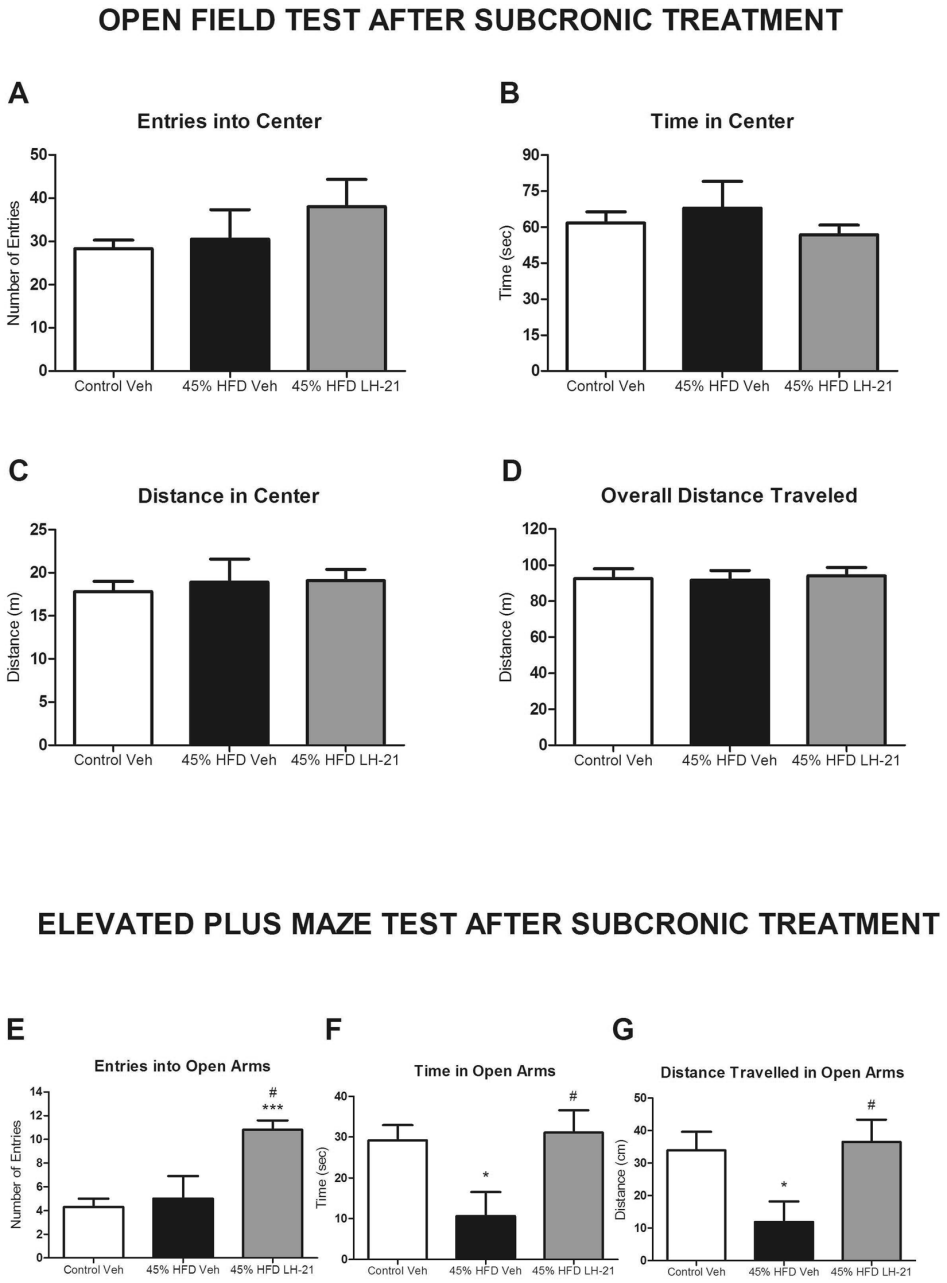
Behavioural study in obese pre-diabetic mice subchronically treated with LH-21. (**A**–**D**) Exploratory activity was recorded for ten minutes in the open field maze in control, 45% HFD-vehicle and 45% HFD-LH-21 mice. Entries into center (**A**), time in center (**B**), distance in center (**C**) and overall distance travelled (**D**) were monitored. No changes among groups were detected. (**E**–**G**) Anxiety was analysed by the elevated plus maze test in these mice. Entries into open arm (**E**), time in open arms (**F**) and distance travelled in open arms (**G**) were recorded for five minutes. 45% HFD-vehicle mice showed an anxiety-like behaviour, with decreased time in open arms and distance travelled in open arms when compared to control mice. Subchronic treatment of obese pre-diabetic mice with LH-21 induced an anxiolytic-like behaviour with increased number of entries into open arms and LH-21 reverted the HFD-induced decrease in time in open arms and distance travelled in open arms. n = 8–10 mice each group. One-way ANOVA and Bonferroni’s post-test, *p < 0.05 *versus* Control-Vehicle, ^#^p < 0.05 *versus* 45% HFD-Vehicle.

Given the conflicting reports on the ability of LH-21 to cross the blood-brain-barrier, and in the context of studying the metabolic actions of LH-21 on obese pre-diabetic mice, we decided to explore the putative actions of this compound in behaviour, at both the acute and subchronic levels. Locomotion and anxiety were assessed in drug naïve obese pre-diabetic mice as well as after acute and subchronic treatment with LH-21 by analyzing performance on the open field test and the elevated plus maze test. No changes in locomotion were detected in obese pre-diabetic mice (Figs 4A–D and S2A–D). However, obese pre-diabetic mice displayed decreased time and distance travelled in open arms during the elevated plus maze test (Figs 4F,G and S2F,G), suggesting the presence of anxiety in these mice. More importantly, acute administration of LH-21 increased both time spent and distance travelled in the center during the open field test performance (Fig. 4B,C), suggesting an anxiolytic effect. In fact, the anxiety traits displayed by obese pre-diabetic mice were totally reverted by acute administration of LH-21 (Fig. 4E–G). No effect of LH-21 after subchronic treatment was found in open field test performance (Fig. 5A–D) in contrast to what was found after LH-21 acute injection (Fig. 4A–D). However, it is noteworthy that subchronic treatment of obese pre-diabetic mice with LH-21 reverted the obesity-induced anxiety, as did acute injection of LH-21, increasing number of entries, time spent and mean distance travelled in open arms when compared to vehicle-injected obese pre-diabetic mice (Fig. 5E–G).

### LH-21 induces behavioural changes in mice through GPR55 receptor

**Figure 6:**
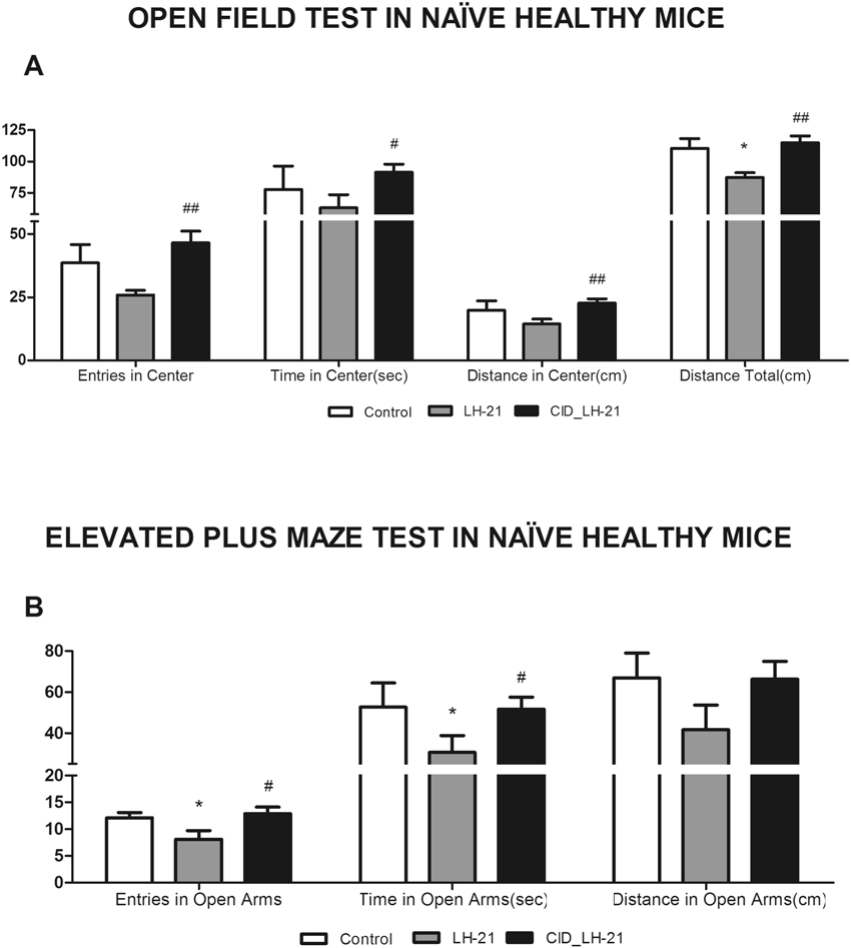
Behavioural study in healthy mice acutely treated with LH-21 and CID16020046. (**A**) Exploratory activity was recorded for ten minutes in the open field maze in Veh-, LH-21- and CID + LH21-injected healthy mice. Entries into center, time in center, distance in center and overall distance travelled were monitored. Acute LH-21 injections tended to decrease exploratory activity in the center, and decreased total exploratory activity. These effects were totally reverted by CID16020046 pre-injection. (**B**) Anxiety was analysed by the elevated plus maze test in these mice. Entries into open arm, time in open arms and distance travelled in open arms were recorded for five minutes. LH-21-injected mice showed an anxiety-like behaviour with decreased entries and time into open arms when compared to control Veh-injected mice. These anxiogenic effects of LH-21 were totally reverted by CID16020046 pre-injection. n = 6–8 mice each group. One-way ANOVA and Bonferroni’s post-test, *p < 0.05 *versus* control; ^#^p < 0.05, ^##^p < 0.01 *versus* LH-21-injected mice.

The ability of LH-21 in counteracting obesity-induced anxiety is apparently mediated through CB1-independent mechanisms, given CB1 blockage is known to promote anxiety. Indeed, we have previous evidence of GPR55 mediating some metabolic actions of LH-21^17^. Therefore, we postulated that central LH-21-driven actions might be mediated through this receptor. To challenge this hypothesis we carried out pharmacological experiments in naïve healthy mice that were injected with 3 mg/kg of LH-21 and 3 mg/kg of the GPR55 antagonist CID16020046. Subsequent behavioural analysis was performed in the open field and the elevated plus maze. LH-21 dosage was selected according to previous works using this compound, as stated above, and CID dosage was selected according to previous experiments performed in our lab. LH-21 decreased entries and time into open arms in the elevated plus maze, suggesting anxiogenesis (Fig. 6B). Indeed, a tendency to decrease distance travelled in open arms in the plus maze as well as less central square activity in the open field test was evident (Fig. 6). In addition, LH-21 decreased total locomotion in the open field test (Fig. 6A). These behavioural effects of LH-21 points to a different response in healthy and obese pre-diabetic mice. However, and interestingly, all these behavioural effects of LH-21 were prevented by administration of the GPR55 antagonist CID16020046, suggesting that central LH-21 actions are mediated through this receptor (Fig. 6).

## Discussion

LH-21 was described as a silent CB1 antagonist with poor brain permeability^10^. This pharmacological profile has led to LH-21 being considered part of the new class of promising peripheral CB1 antagonists^3^. However, its pharmacology is still unclear and its potential role in delaying T2D onset as well as the underlying mechanisms have not been explored yet. Herein we demonstrate that subchronic LH-21 treatment: (1) does not induce body weight loss in a mouse model of obesity and pre-diabetes, (2) improves diabetes risk factors such as glucose handling and tissue inflammation, (3) displays cytoprotective actions on both pancreatic islets and the liver by decreasing apoptosis and macrophage infiltration in these tissues, respectively, which suggests tissue-specific actions that confer resistance in the long-term to the onset of diabetes and steatohepatitis and, (4) conveys the ability to counteract obesity-related anxiety, which could have implications in the management of obesity-induced anxiety and related disorders.

The C57Bl/6J strain of mice used in these studies is prone to develop obesity and metabolic disturbances after being fed a HFD for several weeks^14^. We have maintained this mouse strain on a HFD for 15 weeks, which led to the development of a phenotype mimicking that of human obesity and pre-diabetes. Thus, mice developed obesity, glucose intolerance, insulin resistance, hyperinsulinemia, hyperleptinemia, hypoadiponectinemia, systemic low-grade inflammation (increased levels of the pro-inflammatory cytokines IL-6 and CXCL1), fatty liver, macrophage infiltration in the liver and islets of Langerhans and increased apoptosis in these tissues. These obese pre-diabetic mice were subchronically treated with 3 mg/Kg/day LH-21, a dose that has been previously used in other studies focused on anti-obesity actions of LH-21^12, 13, 15^. We did not find body weight loss or decreased food intake during LH-21 treatment, in contrast to what has previously been reported following LH-21 delivery to rats fed a HFD^13^. This discrepancy might be related to the different animal model (Wistar rats *versus* C57Bl/6J mice) and/or the obesity induction protocol used (60% HFD for ten weeks *versus* 45% HFD for fifteen weeks). However, and in agreement with this previous study, we did not detect normalization in fat content in the liver between LH-21- and Vehicle-treated mice, as assessed by changes in red-oil O staining. Interestingly, despite LH-21 not inducing reductions in food intake and body weight in our model, our results points to LH-21 inducing a metabolic improvement in relevant parameters such as glucose handling, decreased insulin secretion, decreased hyperinsulinemia, decreased HOMA-IR index and a tendency to decrease fasting glucose. In addition, LH-21 treatment also slightly ameliorated the HFD-induced low-grade inflammation, with decreased levels of the cytokines leptin and CXCL1. Furthermore, leptin is also an important metabolic regulator with anorexic and weight-reducing effects whose sensitivity is blunted during hyper-leptinemia (leptin resistance) and is restored when plasma leptin is reduced^18^. Taken together, these findings suggest that LH-21 could confer some degree of protection against diabetes development during obesity. In further support of this hypothesis, we found several cytoprotective actions of LH-21 in liver and pancreatic islets. Specifically, HFD-induced apoptosis was reverted in the islets, and macrophage infiltration was decreased in the liver. LH-21 did not affect the number of repairer M2 macrophages in any tissue, thus suggesting that beneficial actions of LH-21 in the liver might be mediated through downregulation of killer M1 macrophages. In agreement with our findings, lower levels of cleaved caspase-3 have been found in islets of streptozotocin -injected mice after treatment with the CB1 antagonist/inverse agonist AM251^19^ and CB1 in macrophages has also been linked to both islet and liver damage^20, 21^. However, the reason for the differential response between these two tissues is unknown and could be related to the level of expression and/or the specific cell type expressing the target receptor. Interestingly, a recent study with JD5037, a peripheral antagonist/inverse agonist of CB1 receptors, has shown potent and specific actions of this compound at hepatocytes and β-cells by targeting the CB1b isoform^22^, underlining the variety of actions of CB1 antagonists.

An earlier study with AM6545, a neutral CB1 antagonist with reduced brain penetrance, indicated that it was less effective than rimonabant in reducing body weight, adiposity, insulin resistance, and hyperleptinemia, and it had minimal effect on food intake^7^. Later, JD5037 was found to robustly reduce food intake, body weight, and adiposity without targeting brain CB1 receptors^8^. From the above studies, it has been concluded that inverse agonism is a beneficial pharmacological property in peripheral CB1 antagonists for improving metabolism^8^. LH-21 was initially described as a neutral CB1 antagonist with poor brain penetrance and a paradoxically low affinity *in vitro* ^10^, though it was later reported as a brain-permeant, weak CB1 inverse agonist^11^. Our results pinpoints to specific anti-diabetic actions of LH-21 that are probably derived from its ability to antagonize, with lower affinity than other antagonists, the CB1 receptors, also possibly having a role their weak inverse agonism properties. Furthermore, we have recently found that some metabolic actions of LH-21 could be mediated through the cannabinoid-sensitive receptor GPR55^17^ and this receptor is known to be expressed in peripheral metabolic tissues and in the hypothalamus among other brain regions^23–26^, being regulated by nutritional status and other physiological processes involved in energy balance^27^. Indeed, lack of GPR55 has been linked to increased adiposity and insulin resistance^28^. Interestingly, acute food intake experiments with a single high dose of LH-21 (60 mg/kg) in WT and CB1-KO mice have revealed a CB1-independent decrease of overnight feeding and body weight gain^11^. So, it is plausible that, together with CB1-mediated actions, some of the metabolic effects of LH-21 are mediated through GPR55.

Obesity is associated with an increased risk of anxiety in humans^29^. This relationship has also been demonstrated in obese mice fed a HFD^30, 31^, which also displayed functional alterations and neural adaptations in GABAergic dorsomedial hypothalamic neurons and brain reward circuitry, respectively. Previous studies have suggested that LH-21 has lower BBB permeability than other CB1 antagonists, like rimonabant, and that acute effects of LH-21 on feeding are not associated with anxiety-like behaviours^12, 15^. However, there is evidence of LH-21 crossing the BBB to some extent. For example, *i*.*p*. administration of LH-21 was found to slightly decrease ethanol self-administration^12^, LH-21 partially antagonized motor depression induced by central administration of a cannabinoid receptor agonist (CP55940)^12^ and relatively high concentrations of LH-21 were detected in brain following an intravenous injection of the compound^11^. Interestingly, the extent to which LH-21 can modulate central processes such as anxiety has not been explored yet. Here we show that LH-21, whether under acute or subchronic treatment, reduced HFD-induced anxiety behavior by increasing the time and distance travelled in the open arms during the elevated plus maze performance. Moreover, the number of entries into the open arms were also increased in LH-21-treated mice. In the case of acute injections, LH-21 even increased time in the center and distance in the center in the open field test when compared to control mice, thus showing a potent anxiolytic effect in obese pre-diabetic mice. These effects were not due to hyper-locomotion as the overall distance travelled was not affected by the treatment. Taken together, these experiments suggest that LH-21 can cross the BBB in sufficient amounts to induce *per se* behavioural changes, also modulating obesity-induced anxiety. Given that CB1 antagonists have been unequivocally described as anxiogenic drugs and, specifically, they have been found to decrease exploration on the elevated plus-maze^32^ our results suggest that these central effects of LH-21 are not mediated through CB1 receptors. Interestingly, as stated above, we have recently found that some metabolic actions of LH-21 could be mediated through GPR55^17^, and this receptor is also known to be expressed at the central nervous system^25, 26, 33^. Specifically, the nucleus accumbens and the hypothalamus are among the brain regions with higher GPR55 expression^25, 26^, both of them being involved in anxiogenesis^30, 31^. Thus, anxiety-related effects of LH-21 could be mediated through central GPR55 receptors. To challenge this hypothesis we performed a behavioural study in healthy mice acutely treated with LH-21 and the GPR55 antagonist CID16020046. Interestingly, CID16020046 prevented LH-21-induced behavioural changes, thus strongly suggesting a key role of GPR55 in mediating the LH-21-driven changes in anxiety. Nevertheless, LH-21 induced anxiogenesis in healthy mice, an effect that was the opposite to that exerted on obese pre-diabetic mice. Furthermore, LH-21 decreased exploratory activity in healthy mice, in contrast with obese pre-diabetic mice. These opposing effects between healthy and obese pre-diabetic mice could be related to changes in the expression of GPR55 during obesity development, in parallel to the well-known changes that take place in the endocannabinoid system during this disease’s development^34–36^. Indeed, the endocannabinoid system is functionally connected with GPR55^37–39^. Moreover, in agreement with our findings in healthy mice, alterations in physical activity have been reported in lean mice lacking GPR55^28^. Although our results highly support the involvement of GPR55 in central LH-21 actions, a contribution of brain CB1 receptors cannot be ruled out in healthy mice. Therefore, a more in-depth pharmacological evaluation of the LH-21-GPR55 interaction and the signalling pathways potentially involved are warranted.

In conclusion, our study reveals that the compound LH-21 confers body-weight independent improvement in glucose handling in a mouse model of obesity and pre-diabetes, also displaying cytoprotective actions at both pancreatic islets and the liver. Indeed, LH-21 can counteract obesity-related anxiety, probably through GPR55 receptor. Taken together, these findings unravel a potential for LH-21 in delaying diabetes onset as well as for management of obesity-related CNS disturbances.

## Methods

### LH-21 and CID16020046

LH-21 was purchased from Cayman (Cayman Chemical, Ann Harbor, MI), dissolved in 100% ethanol and stored in 30 mg/ml aliquots. For daily injections, fresh dilutions were prepared in vehicle (5% Tween 80 in saline; final ethanol concentration 1%). Either vehicle or LH-21 were *i*.*p*. injected daily for two weeks in the obese pre-diabetic mice that had been fed a HFD for 15 weeks. An effective dosage of 3 mg/Kgday^−1^ LH-21 was used to treat animals as previously described^12^. For the pharmacological experiments in naïve healthy mice LH-21 was prepared and administered as above. CID16020046 was purchased from Tocris (Tocris Bioscience, Bristol, UK), dissolved in 100% DMSO and stored in 30 mg/ml aliquots. In the experiments, CID16020046 was dissolved in saline and *i*.*p*. injected 15 min before LH-21 injections.

### Generation of diet-induced obese pre-diabetic mice and study design

C57BL/6J mice (9 weeks old at arrival) were purchased from Charles River (France) and allowed to acclimatise in the animal facility of IBIMA for one week prior to the experiments. Mice were singly housed under a 12 h light/dark cycle (8:00 pm light off) in a room with controlled temperature (21 ± 2 °C) and humidity (50 ± 10%) and free access to pelleted chow (Standard Rodent Diet A04, SAFE, Panlab, Barcelona, Spain) and water. For induction of obesity and pre-diabetes, groups of ten mice were fed a HFD (D12451 Research Diets Inc, New Brunswick, NJ, USA), containing 45% of Kcal from saturated fat, for 15 weeks (Fig. 1). In parallel, groups of ten age-matched mice were fed a control diet containing 10% of Kcal from fat (D12450 Research Diets Inc). Twice a week, body weight was monitored in awake mice. Glucose handling was assessed by intraperitoneal (*i*.*p*.) glucose tolerance test (GTT) and *i*.*p*. insulin tolerance test (ITT) at weeks 8 and 11, respectively. Behavioural analysis of locomotion and anxiety was performed in naïve obese pre-diabetic mice at week 14 and after acute *i*.*p*. injections of LH-21 [3 mg/Kg, body weight (*b*.*w*.)] at week 15. Inmediately after the 15 week feeding protocol, animals were subchronically treated with LH-21 for two weeks and body weight and food intake were monitored daily. Before euthanasia, a behavioural analysis were again performed. At the end of the study, mice were euthanized by cervical dislocation and tissues immediately collected for further histological and biochemical analysis. The number of mice in each experiment is stated in the figures legends. The European Union recommendations (2010/63/EU) on animal experimentation were followed. All the procedures were approved by the ethic committee of the University of Malaga (authorization no. 2012–0061A).

### Glucose tolerance and insulin sensitivity assessment

During week 8 of the dietary intervention and after LH-21 treatment (week 17), glucose tolerance was assessed by *i*.*p*. GTT. Briefly, following overnight fasting and *i*.*p*. injection of 2 g/Kg D-(+)-glucose (Sigma Aldrich, St Louis, MO) blood glucose was measured from the tail tip at baseline and 15, 30, 45, 60, and 120 minutes after glucose overload using a glucometer (Accu-check, Roche Diagnostic). For insulin sensitivity assessment the *i*.*p*. ITT or the HOMA-IR was used. *i*.*p*. ITT was carried out at week 11 and HOMA-IR after LH-21 treatment. For the *i*.*p*. ITT mice were fasted overnight and then *i*.*p*. injected with 0.5 IU/Kg of insulin (Actrapid®, Novo-Nordisk Pharma SA, Spain). Blood glucose was monitored as stated above. Glucose AUC was calculated from the corresponding graphs for *i*.*p*. GTT by using ImageJ software (U. S. National Institutes of Health, Bethesda, Maryland, USA). For the *i*.*p*. ITT, glucose values were calculated as a percentage of initial blood glucose and the Kitt (the constant of glucose decay) was indeed calculated. The HOMA-IR index was calculated by using the equation: HOMA-IR = insulin(mIU/L) × glucose (mM)/22.5) from plasma insulin and blood glucose levels collected after euthanasia.

### Islets isolation and *in vitro* glucose-stimulated insulin secretion (GSIS) experiments

Mouse islets of Langerhans were isolated by using the collagenase digestion method, as previously described^40^. Islets were then cultured for 20–24 hours in RPMI-1640 medium supplemented with 11 mM glucose (Invitrogen, CA, USA), 2 mM glutamine, 200 IU/ml penicillin, 200 µg/ml streptomycin and 8% fetal bovine serum stripped with charcoal-dextran (Invitrogen). GSIS experiments were performed as previously described^40^. Briefly, islets were first pre-incubated at 37 °C for 2 h in Krebs-bicarbonate buffer solution containing (in mM): 14 NaCl, 0.45 KCl, 0.25 CaCl2, 0.1 MgCl2, 2 HEPES and 3 glucose, and equilibrated with 95% O2: 5% CO2 at pH 7.4. Size-matched islets, five in each well, were seeded in 0.5 mL fresh buffer containing 3 mM glucose or 11 mM glucose and further incubated for 1 h. After incubation, 1% bovine albumin was added to each well, and the plate was cooled at 4 °C for 15 minutes to stop insulin secretion. The media were then collected and stored at −20 °C until insulin measurement by ELISA (Mercodia, Uppsala, Sweden), according to the manufacturer’s instructions.

### Systemic inflammatory markers

Insulin and the adipokines leptin and adiponectin, as well as a panel of pro-inflammatory cytokines were measured in plasma samples from fasting mice treated with LH-21. At the time of euthanasia, whole blood was collected in EDTA-coated tubes and centrifugated at 2,000 × g, 4 °C, for 10 min to obtain plasma. The levels of plasma insulin, leptin and HMW adiponectin were measured with commercial ELISA kits in accordance with the manufacturer’s instructions (insulin kit from Mercodia (Upsala, Sweden), leptin kit from BioVendor (Brno, Czech Republic) and adiponectin kit from Shibayagi Co. Ltd. (Ishihara, Shibukawa, Japan)). The Pro-inflammatory Panel 1 (mouse) Kit (Meso Scale Diagnostics, Rockville, MA, USA) was used for the quantitative determination of IFN-γ, IL-2, IL-5, IL-6, CXCL1, IL-10 and IL-12p70 in a MSD instrument (SECTOR S 600) equipped with multi-array electrochemiluminescence detection technology (Meso Scale Diagnostics).

### Pancreas and liver histopathology

After euthanasia, the entire pancreas and small pieces of liver from mice were fixed by immersion in 4% paraformaldehyde for 48 hours at 4 °C and then paraffin-embedded. Five micrometer thick sections were cut on a sliding microtome and adhered to poly-L-lysine coated microscope slides. For detection of lipid accumulation, liver samples were cut in a cryostat (10 µm), affixed to microscope slides and air-dried at room temperature for 30 min. Detection of lipid droplet was done by Oil red O staining. Briefly, the liver sections were stained in fresh Oil red O for 20 min, rinsed in 70% alcohol, rinsed in distilled water and finally counterstained with hematoxylin for 1 min. The slides were then viewed under a light microscope and the Oil red O stain intensities were quantified from photomicrographs using Image J software. Three photomicrographs were taken from each section, and 4 different sections were analysed from each mouse. A total of four mice from each group were studied. Macrophages infiltration and the presence of the M2 subtype were analysed by immunohistochemistry in islets and liver. Sections were incubated overnight with an antibody against the macrophage marker F4/80 (Abcam, Paris, France), 1/100, or the M2 markers Mrc-1 (Abcam), 1/500, and CD163 (Abcam), 1/500, in 2.5% serum, 0.5% Triton X-100 and 0.01% sodium azide in PBS, rinsed three times with PBS (3 × 5 min), followed by incubation for 2 hours with a secondary antibody, biotin-conjugated rabbit anti-rat polyclonal (DAKO), 1/800 for anti-F4/80, and biotin-conjugated swine anti-rabbit polyclonal (DAKO), 1/3000 for anti-Mrc-1, and 1/500 for anti-CD163. After three washes in PBS, sections were incubated in extravidin-HRP (1/1000) for one hour. Sections were washed again three times and developed with 1% DAB, 0.04% nickel in PBS. The number of F4/80, Mrc-1 and CD163 positive cells was quantified from photomicrographs using Image J software and expressed as cells/mm^2^. The numbers of photomicrographs, sections and mice used for analysis of total and M2 macrophages in the liver and islets were the same as for the Oil red O staining. In pancreases, all islets from each section were analysed. Liver and islet cell apoptosis was assessed using an *in situ* apoptosis detection kit (Roche, Indianapolis, IN, USA) according to the manufacturer’s instructions. TUNEL-positive cells were evaluated by ImageJ sofware and expressed as cell/mm^2^. The numbers of photomicrographs, islets, sections and mice analysed were the same as for the macrophages study. Some slides were counterstained with hematoxylin. As a negative control, the primary antibody was omitted in some slides from each group and under these conditions no immunostaining was detected.

### Behavioural characterization

Mice locomotion and anxiety were evaluated by the open field test and the elevated plus maze, respectively, after the development of obesity and pre-diabetes and following both acute and subchronic administration of LH-21 to these mice. Indeed, these behavioural tests were conducted in naïve healthy mice (12 weeks old) acutely injected with LH-21 and the GPR55 antagonist CID16020046. The GPR55 antagonist was *i*.*p*. injected 15 min before LH-21 injections. Testing took place between 10:00 and 17:00 hours, with equal distribution of testing for control and obese mice throughout the day. The animals were kept in the test room for 30 min prior to the start of the experiments to normalize them to their new environment. For the open field test, each mouse was placed in a dimly illuminated observation cage (109 × 49 × 49 cm) with a clear front panel. Activity in the open field maze was tracked using a digital video camera coupled to the SMART software (PANLAB, Barcelona, Spain). The maze is virtually divided into two areas, centre and perimeter; we recorded the time spent in each area during 10 min periods as well as the number of crosses between areas and the total distance travelled. For the elevated plus maze test each mouse was placed in the central square (5 × 5 cm) facing an open arm, and allowed to explore the maze for 5 min, during which time a video tracking system recorded its behavior. The following variables were analysed: time spent in the open arms, closed arms and central square; open-arm entries and closed-arm entries (both the absolute number and as a percentage of total arm entries) and distance travelled in open arms and closed arms. The maze was thoroughly cleaned with a dry cloth between sessions.

### Data analysis

Data are expressed as mean ± standard error of the mean (SEM). The symmetry of the data was tested by Kolmogorov-Smirnov and Shapiro-Wilk normality tests. The statistical significance of differences in mean values was assessed by unpaired Student’s t-test or analysis of variance (ANOVA), as appropriate for parametric data. Statistical analysis was performed with GraphPad Prism (GraphPad Software, Inc., La Jolla, CA, USA). A p-value < 0.05 was considered significant.

## Electronic supplementary material

Supplementary Figures and legends

## References

[1] WHO. Health in 2015: from MDGs to SDGs. Global status report on non-comunicable diseases. **1**, 133–152 (2014).

[2] Di Marzo V, Després JP (2009). CB1 antagonists for obesity–what lessons have we learned from rimonabant?. Nat Rev Endocrinol.

[3] Bermudez-Silva FJ, Viveros MP, McPartland JM, Rodriguez de Fonseca F (2010). The endocannabinoid system, eating behavior and energy homeostasis: The end or a new beginning?. Pharmacology Biochemistry and Behavior.

[4] Nogueiras R (2008). Peripheral, but Not Central, CB1 antagonism provides food intake-independent metabolic benefits in diet-induced obese rats. Diabetes.

[5] Kunos G, Osei-Hyiaman D, Bátkai S, Sharkey KA, Makriyannis A (2009). Should peripheral CB1 cannabinoid receptors be selectively targeted for therapeutic gain?. Trends Pharmacol. Sci..

[6] Chorvat, R. J. Peripherally restricted CB1 receptor blockers. *Bioorganic Med*. *Chem*. *Lett*. **23** (2013). 10.1016/j.bmcl.2013.06.066 23902803

[7] Tam J (2010). Peripheral CB1 cannabinoid receptor blockade improves cardiometabolic risk in mouse models of obesity. J. Clin. Invest..

[8] Tam J (2012). Peripheral cannabinoid-1 receptor inverse agonism reduces obesity by reversing leptin resistance. Cell Metab..

[9] Klumpers LE (2013). Peripheral selectivity of the novel cannabinoid receptor antagonist TM38837 in healthy subjects. Br J Clin Pharmacol.

[10] Jagerovic N (2004). Discovery of 5-(4-chlorophenyl)-1-(2,4-dichlorophenyl)-3-hexyl-1h-1,2,4-triazole, a novel *in vivo* cannabinoid antagonist containing a 1,2,4-triazole motif. J Med Chem.

[11] Chen RZ (2008). Pharmacological evaluation of LH-21, a newly discovered molecule that binds to cannabinoid CB1 receptor. Eur J Pharmacol.

[12] Pavon FJ (2006). Antiobesity effects of the novel *in vivo* neutral cannabinoid receptor antagonist 5-(4-chlorophenyl)-1-(2,4-dichlorophenyl)-3-hexyl-1H-1,2,4-triazole–LH 21. Neuropharmacology.

[13] Alonso M (2012). Anti-obesity efficacy of LH-21, a cannabinoid CB 1 receptor antagonist with poor brain penetration, in diet-induced obese rats. Br. J. Pharmacol..

[14] Collins S, Martin TL, Surwit RS, Robidoux J (2004). Genetic vulnerability to diet-induced obesity in the C57BL/6J mouse: Physiological and molecular characteristics. Physiology and Behavior.

[15] Pavón FJ (2008). Central versus peripheral antagonism of cannabinoid CB1 receptor in obesity: Effects of LH-21, a peripherally acting neutral cannabinoid receptor antagonist, in Zucker rats. in. Journal of Neuroendocrinology.

[16] Wang C-Y, Liao JK (2012). A mouse model of diet-induced obesity and insulin resistance. Methods Mol. Biol.

[17] Ruz-Maldonado I (2015). Stimulation of insulin secretion by cannabinoid ligands LH-21 and Abn-CBD in mouse and human islets: evaluation of the role of GPR55. Diabetologia.

[18] Enriori PJ (2007). Diet-induced obesity causes severe but reversible leptin resistance in arcuate melanocortin neurons. Cell Metab.

[19] Kim W (2012). Cannabinoids induce pancreatic β-cell death by directly inhibiting insulin receptor activation. Sci Signal.

[20] Jourdan T (2013). Activation of the Nlrp3 inflammasome in infiltrating macrophages by endocannabinoids mediates beta cell loss in type 2 diabetes. Nat Med.

[21] Mai P (2015). Endocannabinoid System Contributes to Liver Injury and Inflammation by Activation of Bone Marrow-Derived Monocytes/Macrophages in a CB1-Dependent Manner. J Immunol.

[22] González-Mariscal I (2016). Human CB1 Receptor Isoforms, present in Hepatocytes and β-cells, are Involved in Regulating Metabolism. Sci Rep.

[23] Romero-Zerbo SY (2011). Role for the putative cannabinoid receptor GPR55 in the islets of Langerhans. J. Endocrinol..

[24] Moreno-Navarrete JM (2012). The L-α-lysophosphatidylinositol/GPR55 system and its potential role in human obesity. Diabetes.

[25] Henstridge CM (2011). Minireview: recent developments in the physiology and pathology of the lysophosphatidylinositol-sensitive receptor GPR55. Mol Endocrinol.

[26] Marichal-Cancino, B. A., Fajardo-Valdéz, A., Ruiz-Contreras, A. E., Méndez-Díaz, M. & Prospéro-García, O. Advances in the Physiology of GPR55 in the Central Nervous System. *Curr*. *Neuropharmacol* (2016). 10.2174/1570159X14666160729155441 PMC5771053 27488130

[27] Imbernon M (2014). Regulation of GPR55 in rat white adipose tissue and serum LPI by nutritional status, gestation, gender and pituitary factors. Mol. Cell. Endocrinol..

[28] A. M (2016). Deletion of G-protein-coupled receptor 55 promotes obesity by reducing physical activity. Int. J. Obes..

[29] Scott KM (2008). Obesity and mental disorders in the general population: results from the world mental health surveys. Int. J. Obes. (Lond).

[30] de Noronha SR (2017). High fat diet induced-obesity facilitates anxiety-like behaviors due to GABAergic impairment within the dorsomedial hypothalamus in rats. Behav. Brain Res..

[31] Sharma S, Fulton S (2012). Diet-induced obesity promotes depressive-like behaviour that is associated with neural adaptations in brain reward circuitry. Int. J. Obes..

[32] Navarro M (1997). Acute administration of the CB1 cannabinoid receptor antagonist SR 141716A induces anxiety-like responses in the rat. Neuroreport.

[33] Sawzdargo M (1999). Identification and cloning of three novel human G protein-coupled receptor genes GPR52, PsiGPR53 and GPR55: GPR55 is extensively expressed in human brain. Brain Res Mol Brain Res.

[34] Engeli S (2005). Activation of the peripheral endocannabinoid system in human obesity. Diabetes.

[35] Blüher M (2006). Dysregulation of the peripheral and adipose tissue endocannabinoid system in human abdominal obesity. Diabetes.

[36] Matias I (2006). Regulation, function, and dysregulation of endocannabinoids in models of adipose and beta-pancreatic cells and in obesity and hyperglycemia. J Clin Endocrinol Metab.

[37] Anavi-Goffer S (2012). Modulation of L-α-lysophosphatidylinositol/GPR55 mitogen-activated protein kinase (MAPK) signaling by cannabinoids. J. Biol. Chem..

[38] Balenga NA (2014). Heteromerization of GPR55 and cannabinoid CB2 receptors modulates signalling. Br. J. Pharmacol..

[39] Martínez-Pinilla E (2014). CB1 and GPR55 receptors are co-expressed and form heteromers in rat and monkey striatum. Exp. Neurol..

[40] Bermudez-Silva FJ (2016). The cannabinoid CB1 receptor and mTORC1 signalling pathways interact to modulate glucose homeostasis in mice. Dis. Model. Mech..

