# Supplementary material for: The cannabinoid ligand LH-21 reduces anxiety and improves glucose handling in diet-induced obese pre-diabetic mice
Source: Sci Rep. 2017 Jun 21;7:3946. doi: 10.1038/s41598-017-03292-w (PMC5479807; doi:10.1038/s41598-017-03292-w)

The cannabinoid ligand LH-21 reduces anxiety and improves glucose handling in diet-induced obese pre-diabetic mice

Silvana Y. Romero-Zerbo[1,2*], Inmaculada Ruz-Maldonado[1,2,3†], Vanesa Espinosa-Jiménez[1†], Alex Rafacho[4], Ana I. Gómez-Conde[5], Lourdes Sánchez-Salido[5], Nadia Cobo-Vuilleumier[6], Benoit R. Gauthier[6], Francisco J. Tinahones[1,7], Shanta J. Persaud[3], Francisco J. Bermúdez-Silva[1,2 *]

Figure S1. Characterization of obese, pre-diabetic C57Bl/6J mice. A) Body weight changes were monitored twice a week in mice fed a high-fat or control diet for 15 weeks. 45% HFD-fed mice displayed higher body weight, B) *i.p.* GTT after 8 weeks on control or HFD; mice were injected with 2g/Kg glucose and blood glucose was monitored before and 15, 30, 45, 60 and 120 minutes after glucose challenge; right insert (bar graph) represents quantification of area under the curve. 45% HFD mice showed glucose intolerance as assessed by increased area under the curve and higher glucose values between 30 and 60 minutes post-injections, C) *i.p.* ITT after 11 weeks on control or HFD. Mice were injected with 0.5U/kg of insulin and blood glucose was monitored as stated above. Glucose values were calculated as a percentage of initial blood glucose and the Kitt (the constant of glucose decay) was indeed calculated. 45% HFD mice displayed insulin resistance as assessed by decreased Kitt and higher relative glucose values between 30 and 60 minutes post-injections; n= 8-10 mice each group. One-way ANOVA (Bonferroni's post-hoc test) and Student's t test, *p<0.05, **p<0.01, ***p<0.001 *versus* control.

Figure S2. Behavioural study in naïve obese pre-diabetic mice. A-D) Exploratory activity was recorded for ten minutes in control and 45% HFD-fed mice. No HFD-induced changes were detected in entries into center (A), time in center (B), distance in center (C) and overall distance travelled (D). E-G) Anxiety was analysed by the elevated plus maze test. Entries into open arm (E), time in open arms (F) and distance travelled in open arms (G) were recorded for five minutes. 45% HFD mice showed an anxiety-like behaviour with decreased time in open arms and distance travelled in open arms. n= 8-10 mice each group. Student's t test, *p<0.05 *versus* control.

Figure S3. M2 macrophages expression in islets of Langerhans and the liver. The expression of M2 macrophages in islets (A-B) and the liver (C-D) was assessed by Mrc-1 (A and C) and CD163 (B and D) immunostaining. Quantification of immunostaining within islets revealed decreased expression of Mrc-1and a tendency of CD163 to decrease in obese pre-diabetic mice, with no differences between HFD-vehicle- and HFD-LH-21-injected mice. Quantification of immunostaining in the liver revealed similar expression of Mrc-1 and CD163 among groups, with no effect of LH-21 in the number of M2 subtype macrophages. The images are representative of islets and livers from four mice each group and four different sections from each tissue; one-way ANOVA and Bonferroni's post-test, *p<0.05 *versus* control.

Supplementary Figure 1

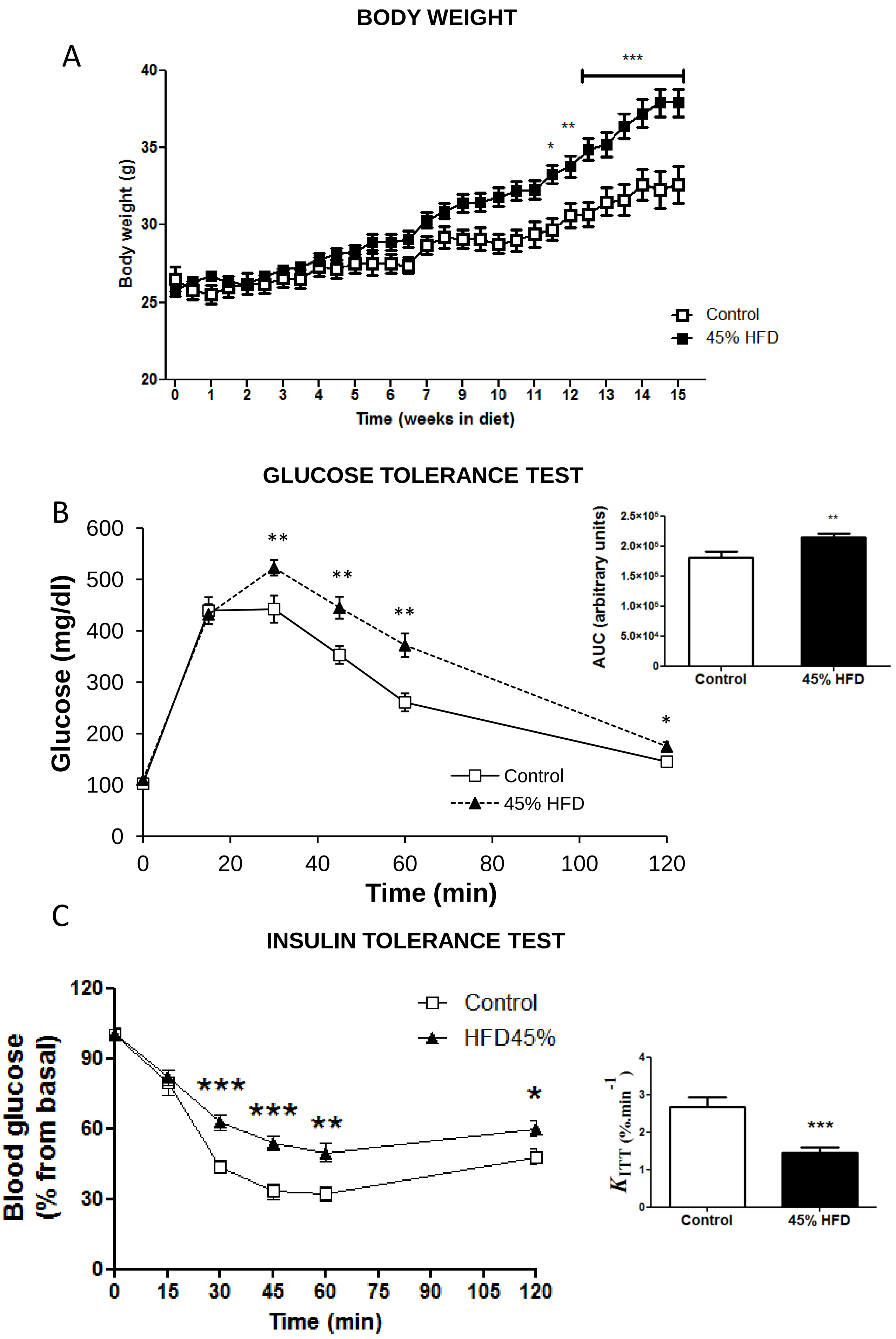

Supplementary Figure 2

OPEN FIELD TEST IN NAÏVE OBESE PRE-DIABETIC MICE

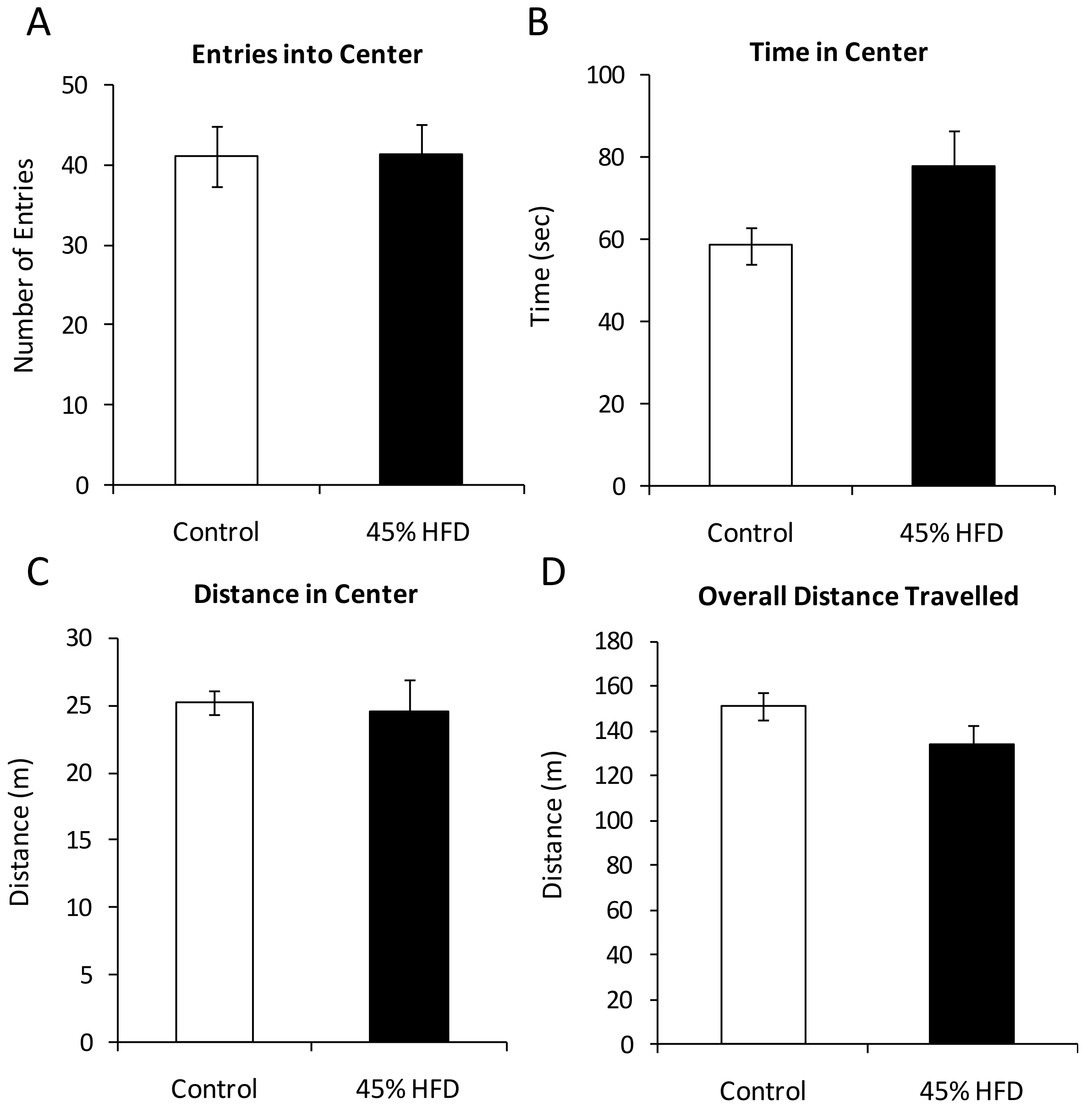

ELEVATED PLUS MAZE TEST IN NAÏVE OBESE PRE-DIABETIC MICE

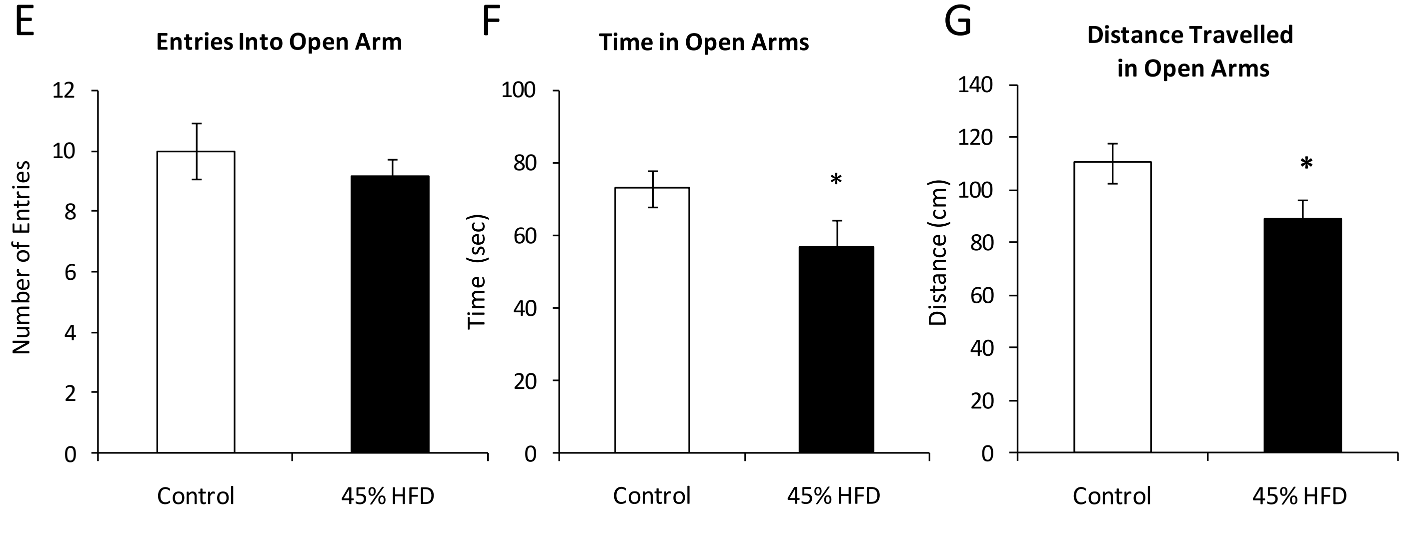

Supplementary Figure 3

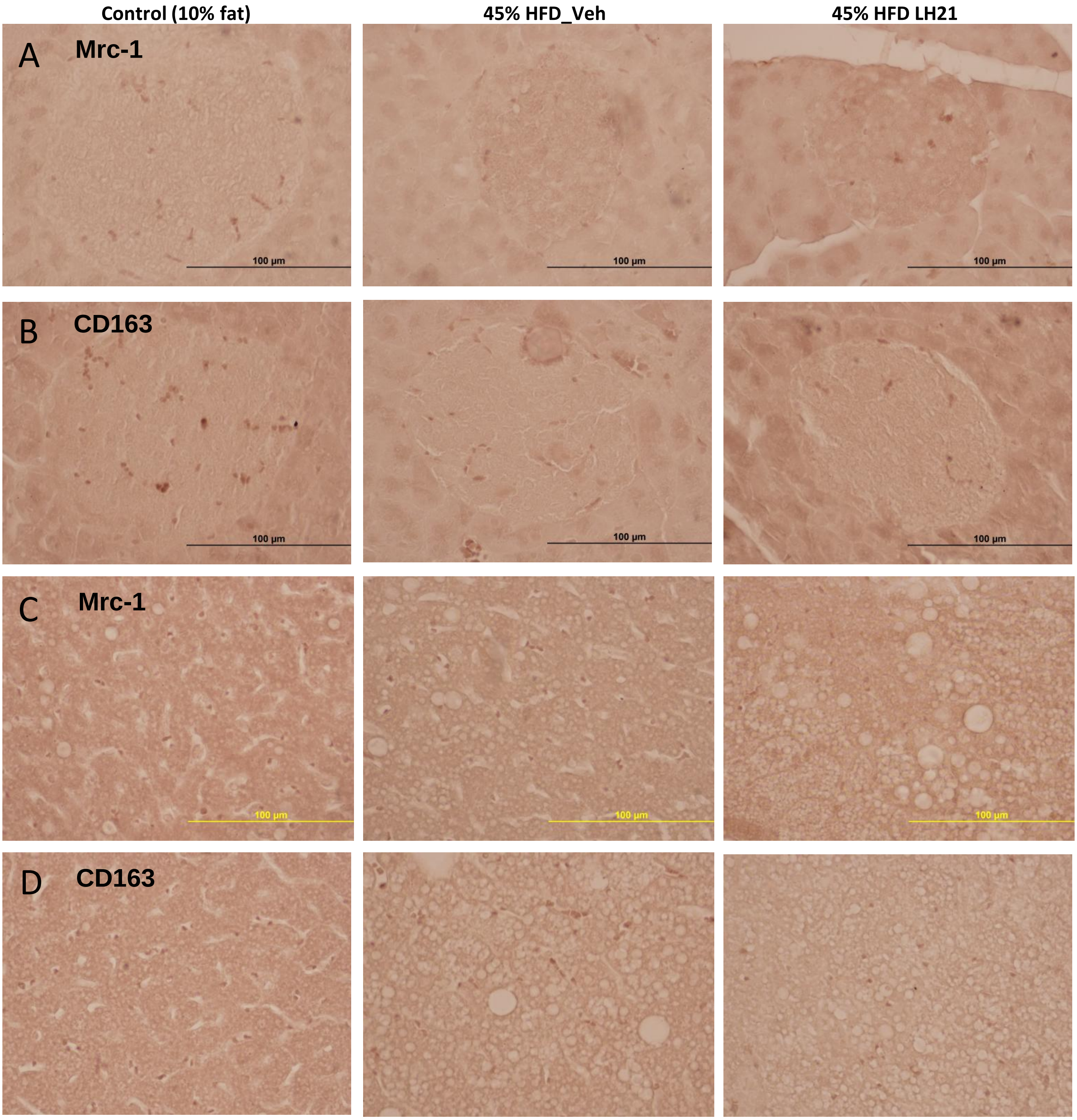

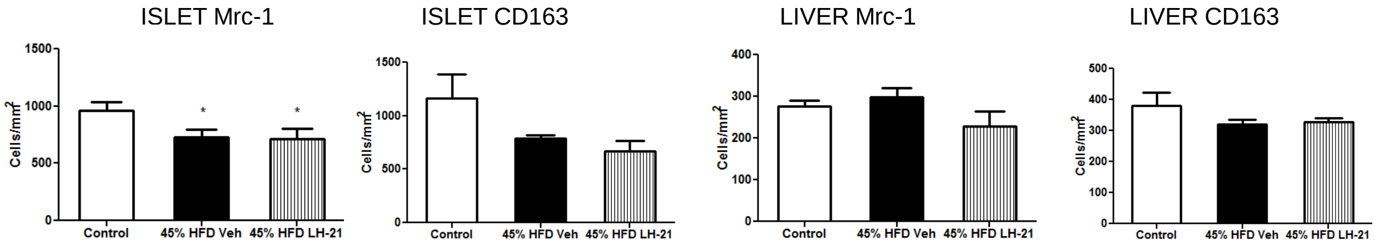

Supplement: Supplementary file 1 — Supplementary Figures and legends [file 41598_2017_3292_MOESM1_ESM.pdf]
